# Supplementary material for: Mental health needs, stressors and coping resources of internally displaced children in post-conflict Syria: A qualitative study with NGO staff
Source: Glob Ment Health (Camb). 2026 Feb 2;13:e25. doi: 10.1017/gmh.2026.10146 (PMC12914474; doi:10.1017/gmh.2026.10146)
Supplement: Çakır-Mete et al. supplementary material [file S2054425126101460sup001.docx]

**INTERVIEW GUIDE**

1. Could you briefly introduce yourself and describe your role in Atmeh Camp?
2. How would you describe Atmeh Camp and the children currently living there?
3. What kinds of challenges do children encounter in their daily lives in the camp?
    Probe (if needed): How do these experiences relate to children’s emotional or psychological well-being?
4. From your perspective, what changes, if any, have occurred in Atmeh Camp since the fall of the Assad regime?
    Probe: How do children appear to experience or respond to these changes?
5. In your view, what are the main mental health needs of children during the post-conflict period?
6. Have children in the camp received news from relatives or family members during this period?
    Probe: How do children seem to respond to such news?
7. How would you describe the uncertainties children face in the post-conflict period?
    Probe: In what ways do these uncertainties influence children’s daily lives or well-being?
8. What plans do families appear to have for the post-conflict period?
    Probe: How do these plans seem to shape children’s experiences or expectations?
9. How do children cope with the challenges they face during the post-regime period?
    Probe: What appears to support their mental or emotional well-being?
10. What role, if any, do religious or cultural beliefs play in how children cope with psychological challenges?
11. How do families and the broader community contribute to children’s ability to cope with psychological difficulties?
12. Are there existing mechanisms or practices within the camp aimed at addressing children’s mental health needs?
     Probe: If so, could you describe how they operate?
13. What types of support or services do you think are needed from organizations working in the region to address these needs?
     Probe: Why do you consider these services important?
